# Synergy analysis of cyanidin-3-O-glucoside and catechin: absorption, transport and lipid metabolism effects

**DOI:** 10.3389/fnut.2025.1637637

**Published:** 2025-08-19

**Authors:** Hangyu Liu, Tienan Xiang, Qianxi Zang, Meihong Liu, Yuying Wang, Wandi Yin, Jingsheng Liu

**Affiliations:** ^1^College of Food Science and Engineering, Jilin Agricultural University, Changchun, Jilin, China; ^2^College of Grain, Oil and Food, Jilin Engineering Vocational College, Siping, Jilin, China; ^3^National Engineering Research Center for Wheat and Corn Deep Processing, Changchun, Jilin, China

**Keywords:** polyphenols, cyanidin-3-O-glucoside, catechin, cholesterol, lipid metabolism, hyperlipidemia

## Abstract

Hyperlipidemia represents a global metabolic epidemic with increasing prevalence, profoundly associated with the etiology of cardiovascular and cerebrovascular diseases. This study investigates the therapeutic potential of two widely distributed bioactive polyphenols, Cyanidin-3-O-glucoside (C3G), catechin, and their synergistic combinatorial formation (C3G-catechin) in modulating hyperlipidemia, using complementary *in vitro* models (Caco-2 monolayer and Caco-2/HepG2 co-culture systems) to simulate intestinal absorption dynamics and lipid metabolic regulation. Our results reveal that the intestinal absorption efficiency follows the order of catechin > C3G-catechin > C3G, primarily mediated through passive diffusion. Furthermore, these polyphenols exhibited significant hypolipidemic effects by downregulating the transcriptional and translational levels of lipid metabolism-related genes, such as SREBP-1, PPARγ, and FAS. This downregulation led to a reduction in key metabolites, including total cholesterol, triglycerides, and LDL-C. Notably, the C3G-catechin combination demonstrated superior regulatory efficacy compared to the individual compounds, suggesting synergistic bioactivity. This study provides mechanistic insights into the enteric transport dynamics and metabolic modulation of dietary polyphenols, highlighting their therapeutic potential to reduce harmful cholesterol level. These findings propose new perspectives for developing nutritional health foods aimed at preventing and treating hyperlipidemia.

## 1 Introduction

Hyperlipidemia is a prevalent metabolic disorder characterized by elevated levels of cholesterol and triglycerides, leading to lipid deposition in arterial walls. This condition can result in atherosclerosis and elevate the risk of cardiovascular disease. The increasing incidence of hyperlipidemia is largely attributed to the global rise in obesity, driven by modern lifestyle changes and unhealthy dietary habits. Traditionally, management of hyperlipidemia involves pharmaceutical interventions and lifestyle alterations, such as dietary control and physical activity. However, some medications may have side effects and tolerance issues that warrant careful consideration.

Polyphenols are a large group of natural antioxidants abundant in plant-based foods and commonly consumed in the daily diet, contributing to protecting against cancer and cardiovascular diseases. Cyanidin-3-O-glucoside (C3G) and catechin are natural bioactive polyphenols associated with the management of hyperlipidemia (1). C3G is reported to reduce LDL-C and increase HDL-C levels, thus improving vascular and lipid metabolism (2–5). Catechin, on the other hand, enhances LDL receptor activity to lower cholesterol absorption and decrease liver cholesterol levels, reducing intra-organ lipid and glucose concentrations (6–8). Understanding the metabolic pathways of these polyphenols in intestinal cells can further inform their application in hyperlipidemia management.

Polyphenol transport significantly affects their bioactivity. The way polyphenols are absorbed, and distributed in the body determines their effectiveness in exerting their beneficial effects. Previous report has shown the absorption and transport mechanisms of polyphenols like C3G and catechin in the small intestine using the Caco-2 intestinal epithelial cell model (9, 10). This model simulates intestinal absorption processes, analyzes metabolites related to lipid metabolism, and evaluates polyphenol absorption (11). The Caco-2/HepG2 co-culture model provides insight into the development of a dual-layer co-culture cells and evaluating the efficacy of the transport and activity of these compounds in reducing lipid levels. This approach helps to explore the transport mechanism and the effect of compounds on regulating lipid metabolism. Despite existing research (12), knowledge gaps remain regarding the absorption and transport of polyphenols from grains and legumes, especially black rice and red beans, necessitating further investigation.

This study aims to evaluate the impact of C3G, catechin, and their combination from black rice and red beans on triglycerides (TG) and total cholesterol (TC) levels, as well as HDL-C, LDL-C, and the expression of lipid metabolism-related genes. The Caco-2 cell monolayer model and the Caco-2/HepG2 co-culture in-vitro model were performed to elucidate their transport types and potential benefits in hyperlipidemia management.

## 2 Materials and methods

### 2.1 Chemicals

Oleic acid (≥ 80% purity, Art. No. O1000) and Fluorescein (≥ 80% purity, Art. No. F6377) were supplied by Aladdin (Shanghai, China). Sodium chloride (Art. No. S9888) was obtained from Beijing Chemical Works. Phosphate-buffered saline (PBS; 1×, Art. No. 10010023) was purchased from Thermo Scientific (Waltham, MA, United States). Fetal bovine serum (FBS, Art. No. 04-001-1ACS) was sourced from BI (Israel), and Dulbecco’s modified Eagle’s medium (DMEM; containing 4.5 g/L D-glucose and 1.5 mM L-glutamine, Art. No. 11995-065) was provided by Gibco (Carlsbad, CA, United States). Antibiotic-Antimycotic (Art. No. 15240062) and 0.25% Trypsin–EDTA (Art. No. 25200056) were acquired from Thermo Scientific. Dimethyl sulfoxide (DMSO, ≥ 99.5% purity, HPLC, Art. No. D2650) and Hanks Balanced Salt Solution (HBSS, Art. No. H6648) were purchased from Sigma-Aldrich (St. Louis, MO, United States). PMSF (Cat No. 36978) were purchased from Thermo Scientific. The triglyceride (TG) assay kit (Art. No. E1003) was supplied by Zhejiang Dongou Diagnostics Co., Ltd., and the low-density lipoprotein cholesterol (LDL-C) assay kit (Art. No. E1015) was provided by Nanjing Jiancheng Bioengineering Institute. Reverse transcription reagents, qPCR fluorescent dyes, and BCA protein assay kits were acquired from Sigma-Aldrich, Solarbio, and Bioengineering Co., Ltd., United States. Primers for PPARγ, Fabp4, Plin1, Srebp1-c, and Fasn were supplied by Jilin Cool Me Biotech Co., Ltd. The following antibodies were used in Western blot: SREBP1 (ABclonal; Cat: A15586; Dilution: 1:1000), FASN (ABclonal; Cat: A19050; Dilution: 1:1000), PPARγ (ABclonal; Cat: A19676; Dilution: 1:2000), FABP4 (ABclonal; Cat: A25792; Dilution: 1:3000), PLIN1 (ABclonal; Cat: A16295; Dilution: 1:3000), and *β*-Actin (ABclonal; Cat: AC038; Dilution: 1:10000).

### 2.2 Cell culture and MTT assay

Cell culture: HepG2 and Caco-2 cells were cultured in a DMEM medium supplemented with 10% fetal bovine serum, 50 U/mL penicillin, and 50 μg/mL streptomycin at 37°C in a humidified condition of 5% CO_2_ incubator (BB16UV, Heraeus, Germany).

MTT assay: Caco-2 cells (5 × 10^4^ cells/well) and HepG2 cells (6 × 10^4^ cells/well) were cultured in 96-well plates for 24 h. Cells were treated with different concentrations (7.5 to 100 mM) of C3G, catechin, and C3G-catechin for 24 h. Subsequently, the supernatant was removed and 200 μL of MTT solution (0.1 mg/mL) was added to each well to incubate for an additional 4 h (200 μL for Caco-2 cells, 100 μL for HepG2 cells, both at a concentration of 0.1 mg/mL). Finally, formazan crystals were completely dissolved in DMSO in a shaker for 10 min and the absorbance was measured at a wavelength of 490 nm using a microplate absorbance reader (FLUOstar Omera, BMG Labtech, Germany). Cell viability was determined using the following equation:

Cell viability (%) = (OD sample/OD control) × 100, where OD is the optical density. The absorbance values for drug fractions (C3G, catechin, and C3G-catechin) were compared to those of untreated culture (control). Each treatment was set up in triplicate.

### 2.3 Cell model construction and metabolite determination assay

#### 2.3.1 Cell monolayer and transport studies

The cells were seeded at a density of approximately 10^5^ cells/cm^2^ on TranswellTM filter (Corning Inc., Lowell, MA) and cultured for 21 d before use. Cell monolayer integrity was controlled by the measurement of trans-epithelial electrical resistance (TEER) with an EVOM epithelial voltammeter equipped with an electrode (World Precision Instruments, Sarasota, FL, United States). Cell monolayers (Caco-2 and HepG2 cells) with TEER values greater than 500 *Ω*·cm^2^ were used in transport assays.

Transport assay: The monolayers were pre-incubated at 37°C for 30–40 min with 5% CO_2_ in a pre-warmed media, which contained Hanks Balanced Salt solution (HBSS, BioWhittaker), HEPES buffer and D-glucose. For the transport of apical (AP) side to basolateral (BL) side, the media was removed after pre-incubation and 0.5 mL of polyphenols was added to the AP side. 1.5 mL of HBSS were added to the BL side. Then 200 μL of samples were taken from the BL side at 30, 60, 90, 120 and 150 min, respectively. The BA sample volumes removed were therewith replenished with same volumes of the HBSS solution. For the transport of BL to AP, polyphenols were added to BL and samples were taken from AP at a series of time points as above mentioned. The TEER was measured from the beginning to the end of the assay.

#### 2.3.2 Caco-2/HepG2 co-culture model

Caco-2 and HepG2 cells were cultured separately at 37°C with 5% CO_2_ in DMEM medium containing 10% fetal bovine serum, 50 U/mL penicillin, and 50 μg/mL streptomycin. Caco-2 cells were cultured in 96-well plates for 24 h and harvested with trypsin–EDTA to seed to the AP side (1 × 10^5^ cells/mL, 500 μL) in monolayers. 1,500 μL of Minimum Essential Medium (MEM) was simultaneously added to the BL compartment. HepG2 cells were seeded to the BL compartment at the day 15. The total culture period was 18 d.

#### 2.3.3 Metabolite determination

For high fat HepG2 model: HepG2 cells were cultured for 24 h and 1 mM oleic acid in DMEM with 10% FBS was added to culture for another 24 h at 37°C (13). Afterwards, cells were harvested and lysed. The supernatant of the lysate was collected to evaluate the change in TG and LDL-C. DMEM with 10% FBS was as control.

For the lipid content assay: The cells were treated with 1 mM oleic acid (OA). After co-incubation for 24 h with final concentrations of 6.25, 25, and 50 μM of polyphenols (C3G, catechin, and C3G-catechin) respectively, cells were stained with Oil Red O for 20 min and washed three times with PBS buffer. The absorbance value was recorded at 530 nm using a microplate absorbance reader. Isopropanol solvent was used as a control (14).

For TG and TC assay: The cells were cultured for 24 h as described above, the model group was treated with 50 μM of C3G, catechin, and C3G-catechin, respectively, for 24 h. The medium was removed and the cells were washed twice with PBS. The levels of triglycerides (TG) and total cholesterol (TC) in the cells were determined according to the instructions of test kits.

### 2.4 Absorbtion-model of C3G, catechin, and C3G-catechin in the Caco-2 cell

Caco-2 cells (1×10^5^ cells/well) were cultured in 96-well plates for 24 h until differentiation, with a TEER value exceeding 400 *Ω*·cm^2^. Bidirectional transport experiments from AP to BL or from BL to AP were performed as mentioned in the transport assay. The apparent permeability coefficients, cumulative release and initial concentrations and efflux rates were determined. In brief, the total transport time was set to 150 min. For AP to BL transport, 0.5 mL of polyphenols were added to AP with 1.5 mL of HBSS to the BL side. 200 μL of samples were collected from BL at different time points (30, 60, 90, 120, and 150 min). For BL to AP transport, polyphenols were added to BL and samples were collected from AP as the mentioned above. The absorption rate of anthocyanins (C3G, catechin or C3G-catechin) from AP to BL was calculated using Equation a as shown:


(a) $$R(\%)=\frac{C_{\mathrm{BL}}\times V_{\mathrm{BL}}}{C_{\mathrm{AP}}\times V_{\mathrm{AP}}}\times 100\;\;$$

In this, R represents the absorption rate (as a percentage); CBL is the anthocyanin concentration of the BL side (in μM); VBL is the volume of anthocyanins from the BL (in L); CAP is the initial anthocyanin concentration of the AP side (in μM), and VAP is the volume of anthocyanins in the AP chamber (in L). The absorption rate of anthocyanins from BL to AP is calculated using the following Equation b:


(b) $$R(\%)=\frac{C_{\mathrm{AP}}\times V_{\mathrm{AP}}}{C_{\mathrm{BL}}\times V_{\mathrm{BL}}}\times 100\;\;$$

In this, R represents the absorption rate (as a percentage); CAP is the anthocyanin concentration from the AP side (in μM); VAP is the volume of anthocyanins in the AP chamber (in mL); CBL is the initial concentration from the BL side (in μM) and VBL is the volume of anthocyanins in the BL chamber (in L).

### 2.5 Detection of lipid metabolism, gene expression levels and relative proteins

#### 2.5.1 Evaluation of lipid-lowering function of metabolites based on the Caco-2/HepG2 co-culture model

The co-culture cells were collected by the previous method from the above mentioned, and the cells were lysed on ice for 30 min using IP cell lysis buffer (20 mM Tris pH 7.5, 200 mM NaCl, 10% glycerol, 0.2 mM DTT, 0.1 mM EDTA, 1 mM protease inhibitor). The contents of TG, T-CHO, LDL-C, HDL-C, AST, and ALT from HepG2 were measured following the test kit instructions. Intracellular protein content was measured to calibrate the levels of relevant metabolites using the BCA Protein Quantitation Kit (Beyotime Biotechnology).

#### 2.5.2 mRNA level detection

Total HepG2 cell RNA was extracted using Trizol reagent (Dalian Baosheng Biological Engineering Co., Ltd.) according to the manufacturer’s instructions, and the concentration was measured by spectrophotometry. RNA reverse transcription was completed by a reverse transcription instrument (Mastercycler gradient, Eppendorf, Germany). Then, the measurements were conducted using a fluorescent quantitative PCR instrument (Agilent Stratagene Mx300P, Agilent, United States), by thermal cycling program steps: 95°C for 30 s denaturation; 40 cycles: 95°C for 10 s denaturation, 60°C for 30 s annealing, 72°C for 30 s extension. *β*-actin was used as a control for normalizing gene expression. The results were represented as fold-change calculations (2^-∆∆Ct^) manually. Primers with sequences were listed in Supplementary Table S1 (15).

#### 2.5.3 Western blot analysis

Cells from the co-culture model were lysed and supernatants were collected. The protein concentrations were determined using the BCA Protein Quantitation Kit. Then electrophoresis of samples was conducted using a SDS-PAGE kit (Beyotime Biotechnology) following the manufacturer’s instructions and transferred to poly-vinylidene difluoride (PVDF) membrane. Next, membranes were incubated in 5% nonfat milk to block non-specific antibody binding for 30 min at room temperature and followed by incubating with primary antibodies and secondary antibodies. Chemiluminescence based-images of the blots were acquired using ImageQuant LAS 500 system (GE, United States). Protein bands were quantified using ImageJ (version 1.53a) software. The changes in expression levels of these proteins detected finally are used to evaluate the sample’s effect on lipid metabolism.

### 2.6 Data analysis

Data analysis was performed using SPSS 20.0 software. All results are presented as the mean ± standard deviation (SD). Statistical significance of differences between groups was determined using one-way analysis of variance (ANOVA) with Tukey’s *post-hoc* test. Significance levels were set at *p < 0.05, **p < 0.01, ***p < 0.001, and ****p < 0.0001.

## 3 Results

### 3.1 Cell viability

C3G and catechin are widely found in plants and fruits and have been shown to act on various diseases, including dyslipidemia and hyperlipidemia (1). Previously we characterized how C3G and catechin inhibit pancreatic lipase, an enzyme essential for fat digestion and absorption (16). However, their roles in absorption and transport remain within the body remain insufficiently understood. To explore this, we employed *in vitro* Caco-2 and HepG2 cell model to simulate polyphenol transport and uptake, aiming to uncover their underlying mechanisms affecting human digestive and liver metabolism. In monitoring the transepithelial electrical resistance values in Caco-2 cells, we initially observed low resistance (~150 *Ω*·cm^2^) attributed to reduced cellular activity post-trypsin digestion. Notably, there was a marked increase as the cells rapidly proliferated and differentiated into intestinal epithelial cells over 3 days. Over the span of 12 days, resistance values progressively rose to a peak 600 Ω·cm^2^, after which they stabilized for 6 days. This stability indicates full cellular differentiation, characterized by enhanced epithelial integrity and reduced cell proliferation.

**Figure 1:**
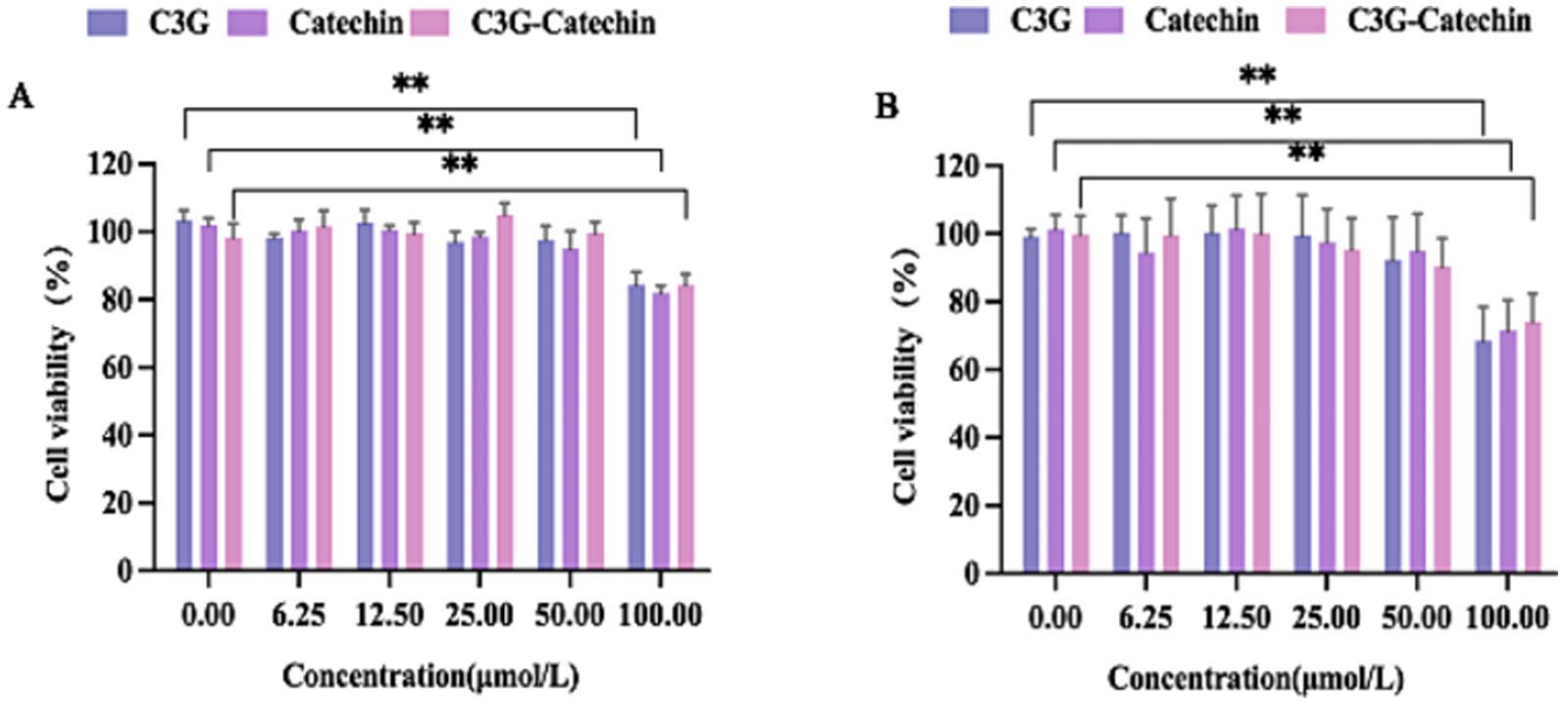
The characteristics of polyphenols transport across Caco-2 cell and HepG2 cell monolayers. **(A)** The effect of different concentrations of polyphenols on the activity of Caco-2 cells. **(B)** The effect of different concentrations of polyphenols on the activity of HepG2 cells. Values are means + SD. *p < 0.05; **p < 0.01. Data were obtained from Student’s *t* test.

Given the reported potential toxicity of certain polyphenols, we investigated the cytotoxicity of C3G and catechin in Caco-2 cells by measuring cell viability across a concentration range (0, 6.25, 12.5, 25, 50 and 100 μM, respectively), as depicted in Figure 1A. No significant changes in cell vitality were observed at concentrations below 50 μM. However, viability was reduced to 80% at 100 μM compared to the control (0 μM), indicating notable cytotoxicity. Consequently, 50 μM was selected for further small intestinal transport studies in Caco-2 cells. Parallel assays were performed in HepG2 cells to optimize the concentration (Figure 1B). HepG2 cells demonstrated comparable cytotoxicity at 100 μM, with no significant effects at 50 μM (1B). Accordingly, 50 μM was also chosen for the transport studies in subsequent assays.

### 3.2 Transmembrane transport of polyphenols in Caco-2 cell mode

**Figure 2:**
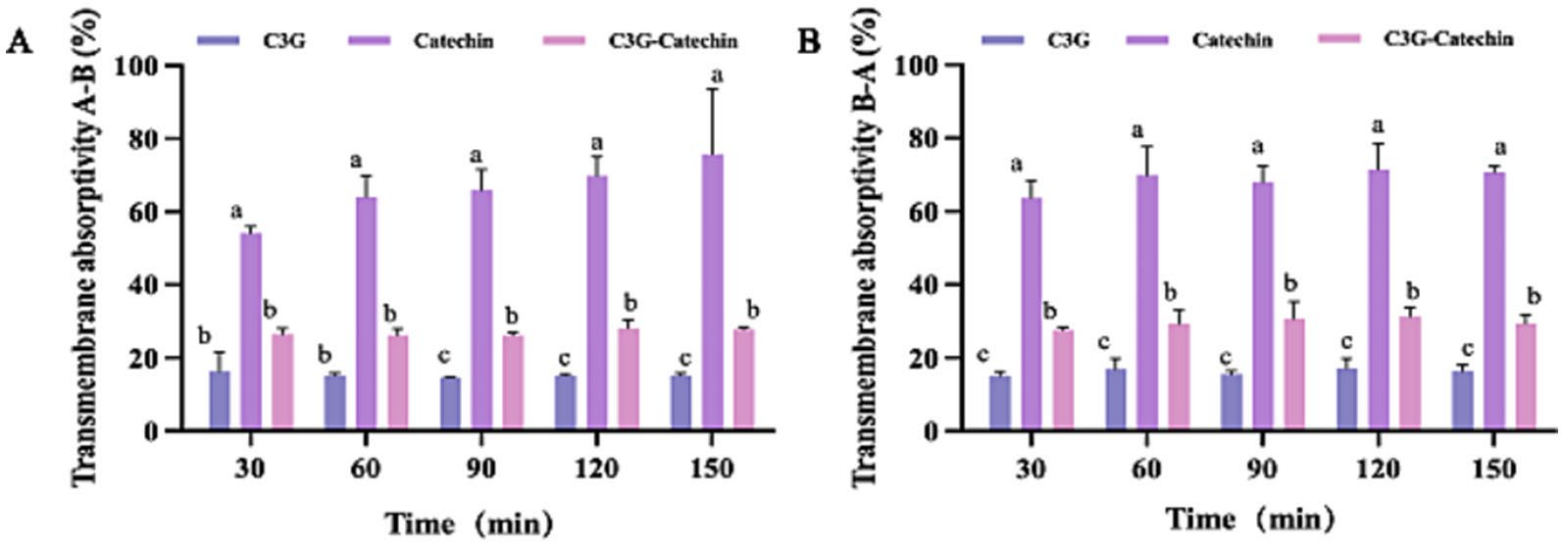
Bi-directional transport of polyphenols in Caco-2 cells. Transport absorptivity of polyphenols from the **A** to the **B** side **(A)** or from the **B** to the **A** side **(B)**. Caco-2 cell monolayers were incubated at 37°C in HBSS with polyphenols at five time points (30, 60, 90, 120 and 150 min). Different letters indicate the significant differences in the same polyphenols at different time points (*p < 0.05).

To further evaluate the transport of three types of polyphenols in Caco-2 cells, we measured their absorption rates and apparent permeability coefficients (Papp). The Papp is often used to assess the transport of chemical entities or active pharmaceutical ingredients across biological barriers and indicates their bioavailability within the body. As shown in Figure 2A, the absorption rates of catechin and C3G-catechin combination significantly increased over time (measured at 30, 60, 90, 120 and 150 min, respectively) at constant concentrations on the BL side. In contrast, the absorption rate of C3G remained relatively stable at 22.47% throughout the study. Catechin exhibited the highest absorption rate, reaching 73.41% within 150 min, while C3G-catechin reached 30.19%. These differences in absorption rates were attributed to the number of hydroxyls groups present in the polyphenols, where a higher number of hydroxyl groups corresponded to lower absorption rates. Indeed, upon analyzing the hydroxyl content of the polyphenols, we found that C3G contains eight hydroxyls, whereas catechin has five, which correlates with the higher absorption rate observed for catechin. The trend of efflux rates on the BL side mirrored that on the AP side. Specifically, the efflux rates for C3G, catechin and C3G-catechin were 15.41, 68.71 and 26.84%, respectively (Figure 2B). Among those, catechin demonstrated a distinct efflux rate compared to the other two polyphenols. These data indicate that different polyphenols exhibit varying absorption and efflux rates.

To study the membrane structure and efflux functionality of Caco-2 cells, we used fluorescein, a permeability marker for characterizing membrane transport in a transmembrane transport assay as shown in Table 1. The Papp value for fluorescein in our transport model, ranging from 0.48–0.58 × 10^−6^ cm/s, was consistent with the permeability grades of the Caco-2 model (17), further indicating that the cells in the transport assay were in good condition with intact membranes and functional efflux systems. Together, these data support the transport model’s suitability for simulating the metabolism of compound absorption and transport *in vitro*.

**Table 1:** The effect of different time and P-GP inhibitors on bidirectional drug transport in Caco-2 cell model.

Concentration/50 μmol/L		30 min	60 min	90 min	120 min	150 min
Papp (AP → BL)/ (×10^−5^ cm /s)	Fluorescin	5.75 ± 0.17	5.07 ± 1.72	4.59 ± 2.21	4.6 ± 2.63	4.65 ± 2.94
	C3G	3.9 ± 1.32^Aa*^	1.8 ± 0.07^Cb*^	1.1 ± 0.02^Cb*^	0.9 ± 0.03^Cb*^	0.7 ± 0.03^Bb*^
	catechin	7.9 ± 0.26^Ba^	4.6 ± 0.43^Ab*^	3. ± 0.27^Ac*^	2.5 ± 0.19^Ad*^	2.2 ± 0.52^Ad*^
	C3G-catechin	4.9 ± 0.29^Ba*^	2.4 ± 0.16^Bb*^	1.6 ± 0.05^Bc*^	1.3 ± 0.11^Bd*^	1.0 ± 0.02^Bd*^
	C3G + Prob	8.47 ± 0.39	4.55 ± 0.09	3.12 ± 0.02	2.45 ± 0.03	1.83 ± 0.09
	catechin+Prob	9.19 ± 0.53	4.46 ± 0.36	3.15 ± 0.14	2.48 ± 0.19	1.93 ± 0.49
	C3G-catechin+Prob	8.56 ± 0.42	4.37 ± 0.29	3.15 ± 0.27	2.41 ± 0.13	1.97 ± 0.53
	C3G + Ver	11.4 ± 0.49	5.73 ± 0.21	4.05 ± 0.12	3.16 ± 0.17	2.47 ± 0.14
	catechin+ Ver	11.2 ± 0.23	6.02 ± 0.37	3.98 ± 0.31	3.25 ± 0.52	2.54 ± 0.17
	C3G-catechin+ Ver	10.5 ± 0.39	6.14 ± 0.27	4.21 ± 0.52	3.48 ± 0.27	2.78 ± 0.25
Papp (BL → AP)/(×10^−5^ cm /s)	Fluorescin	3.2 ± 0.05	1.57 ± 0.15	1.12 ± 0.13	0.87 ± 0.1	0.7 ± 0.08
	C3G	1.8 ± 0.14^Ba^	1.0 ± 0.17^Bb^	0.6 ± 0.04^Cc^	0.5 ± 0.08^Ccd^	0.4 ± 0.04^Cde^
	catechin	6.1 ± 2.78^Aa^	2.5 ± 0.28^Ab^	1.6 ± 0.11^Ab^	1.31 ± 0.13^Ab^	1.0 ± 0.03^Ab^
	C3G-catechin	2.5 ± 0.06^Ba^	1.3 ± 0.18^Bb^	0.9 ± 0.15^Bc^	0.73 ± 0.06^Bd^	0.5 ± 0.04^Bd^
	C3G + Prob	5.31 ± 0.72	2.79 ± 0.37	1.62 ± 0.08	1.29 ± 0.09	1.06 ± 0.04
	catechin+Prob	5.36 ± 0.52	2.50 ± 0.27	1.64 ± 0.37	1.27 ± 0.27	0.98 ± 0.34
	C3G-catechin+Prob	6.58 ± 0.34	3.24 ± 0.45	1.77 ± 0.28	1.44 ± 0.39	1.66 ± 0.23
	C3G + Ver	5.49 ± 0.66	3.07 ± 0.2	2.09 ± 0.03	1.71 ± 0.15	1.21 ± 0.28
	catechin+Ver	6.38 ± 0.37	3.45 ± 0.29	2.05 ± 0.56	1.68 ± 0.29	1.32 ± 0.34
	C3G-catechin+Ver	6.79 ± 0.28	3.34 ± 0.42	2.11 ± 0.08	1.95 ± 0.18	1.75 ± 0.38
ER	Fluorescin	0.56	0.34	0.3	0.27	0.25
	C3G	0.49	0.56	0.53	0.57	0.54
	catechin	0.77	0.55	0.52	0.51	0.48
	C3G-catechin	0.52	0.56	0.58	0.56	0.53
	C3G + Prob	0.63	0.61	0.52	0.53	0.56
	catechin+Prob	0.58	0.56	0.52	0.51	0.48
	C3G-catechin+Prob	0.77	0.74	0.56	0.6	0.86
	C3G + Ver	0.48	0.54	0.51	0.54	0.51
	catechin+Ver	0.57	0.57	0.51	0.52	0.52
	C3G-catechin+Ver	0.65	0.54	0.5	0.56	0.65

^a, b, c, d^Indicate significant differences in the same anthocyanin at different time points. If two data points in the same column have different lowercase letters, it means there is a significant statistical difference between these data (*p* < 0.05).

^A, B, C^Indicate significant differences between different anthocyanins at the same time point. If different anthocyanins in the same row have different uppercase letters, it means there is a significant statistical difference in the transport properties between these anthocyanins at the same time point (*p* < 0.05). * Indicate significant differences between anthocyanins in the same transport direction (*p* < 0.05).

Subsequently, the relationship between Papp and permeability in Caco-2 cells was assessed at varying time points (30, 60, 90, 120 and 150 min). As summarized in Table 1, the trend of Papp was consistent with the previously observed absorption rate, decreasing with longer incubation times. The polyphenols exhibited higher Papp values from the AP side to the BL side, compared to the reverse direction (efflux detected from the BL side to the AP side). The type of transport for polyphenols in the small intestine was inferred from the efflux rate (ER), which is the ratio of Papp (AP to BL) to Papp (BL to AP). An ER less than 1, with no significant efflux, suggests that polyphenols can be absorbed via the apical membrane transporter of Caco-2 cells and may primarily function via passive diffusion. These results also revealed that the Papp values for all polyphenols from AP to BL were lower than those observed with fluorescein treatment, implying reduced intestinal permeability and oral bioavailability (18).

To directly probe transporter involvement, we performed bidirectional assays with two classical ATP-binding-cassette (ABC) transporter inhibitors: verapamil (100 μM, a potent P-gp inhibitor, abbreviated as ver) and probenecid (1 mM, an MRP inhibitor, abbreviated as Prob). As summarized in Table 1 (VER and PROB rows), inhibiting these transporters did not alter the absolute Papp values and the corresponding ER values remained under 1 and were not significantly different from the control groups (*p > 0.05*, *n* = 3). In addition, the Papp value decreased with time. These data confirm that C3G, catechin and C3G-catechin are not substrates of P-gp or MRP2, suggesting passive diffusion as the dominant transport mechanism.

Consequently, our inhibitor study provides direct mechanistic evidence that polyphenols such as C3G, catechin and C3G-catechin are transported into the small intestine via passive diffusion in a time-dependent manner.

### 3.3 Lipid droplets visualization by oil red O staining and metabolite analysis in high-fat HepG2 cells

**Figure 3:**
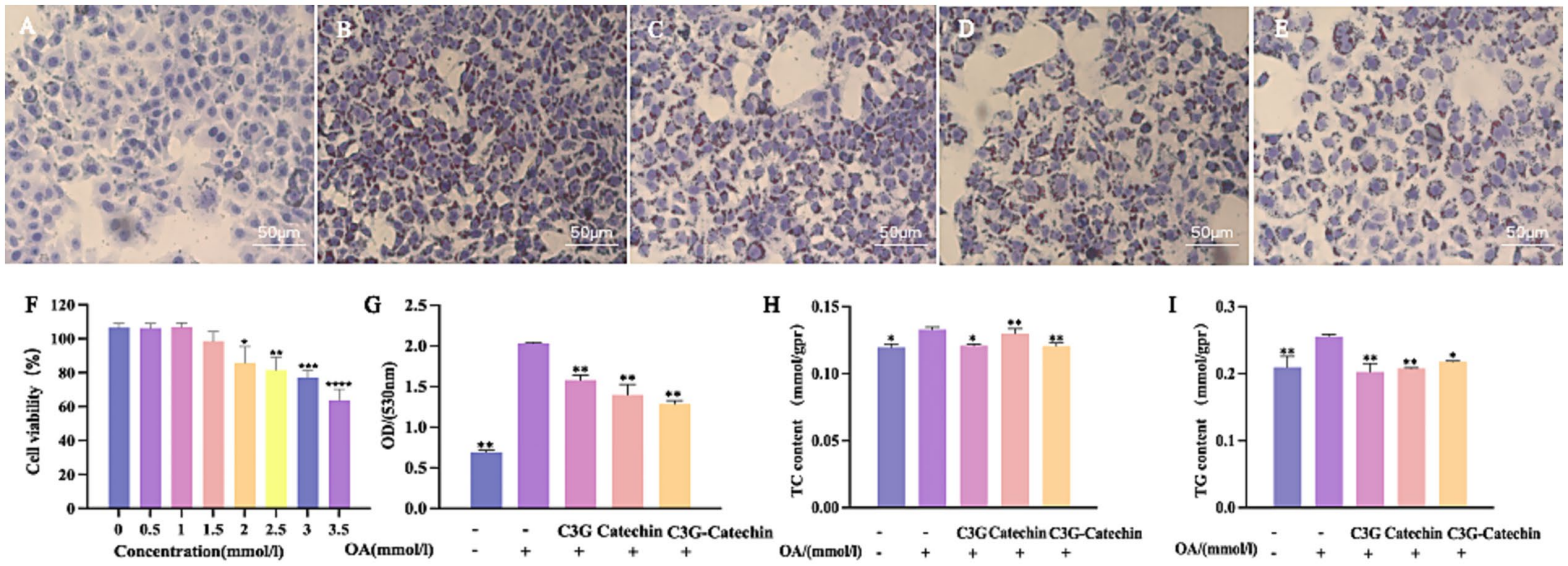
Polyphenols reduced lipid accumulation in HepG2 cells. Representative images of Oil Red O staining depicting lipid droplets in HepG2 cells: **(A)** Normal control; **(B)** OA-induced model; OA-induced cells treated with **(C)** 50 μM C3G, **(D)** 50 μM catechin, or **(E)** 50 μM C3G-catechin combined treatment, respectively. Treatment with C3G, catechin, or their combination effectively suppressed OA-induced lipid accumulation. **(F)** Cell viability under different compound concentrations. **(G)** Quantification of lipid content at a wavelength of 530 nm. ELISA assay for TC **(H)** and TG **(I)** in cells of untreated, OA-induced and compound-treated OA-induced HepG2 cells. Values are means + SD. *p < 0.05; **p < 0.01; ***p < 0.001; ****p < 0.0001. Data were obtained from Student’s *t* test.

To investigate the effects of C3G and catechin on intracellular lipid accumulation and metabolism, we established a high-fat model using HepG2 cells. As shown in Figure 3A, the control group exhibited some lipid droplets accumulation, as identified by Oil Red staining (19). In contrast, treatment with 1 mM OA led to a marked increase in lipid droplets accumulation (Figure 3B). Interestingly, cell viability remained unchanged at OA concentrations up to 1.5 mM (Figure 3F), confirming that the model was not cytotoxic under these conditions. When various polyphenols were applied to the cells, the OA-induced accumulation of lipid droplets was reduced (Figures 3C–E,G). Specifically, lipid absorption rates decreased by 22.35, 31.24 and 36.75% for C3G, catechin and the C3G-catechin combination, respectively. These reductions suggest that the polyphenols inhibited lipid droplets accumulation effectively. Given the known effects of the polyphenols on lipid reduction, we investigated potential changes in lipid metabolism. We measured TG and TC content in the high-fat model, as these are key metabolites derived from free fatty acids in various species. In our high-fat model, TG and TC levels increased by 22 and 12%, respectively, compared to the control group (Figures 3H,I). Notably, polyphenol treatment significantly mitigated these elevations (Figures 3H,I), the C3G-catechin group and C3G alone reduced TC and TG contents by 10 and 21%, respectively, when compared to the control, indicating a potent inhibitory effect on TG accumulation in high-fat cells. These findings collectively suggest that polyphenols reduced the accumulation and metabolism of fat acids.

### 3.4 Analysis of lipid metabolism-related protein and gene expression levels

#### 3.4.1 The impact of anthocyanins on lipid metabolism and liver health

**Figure 4:**
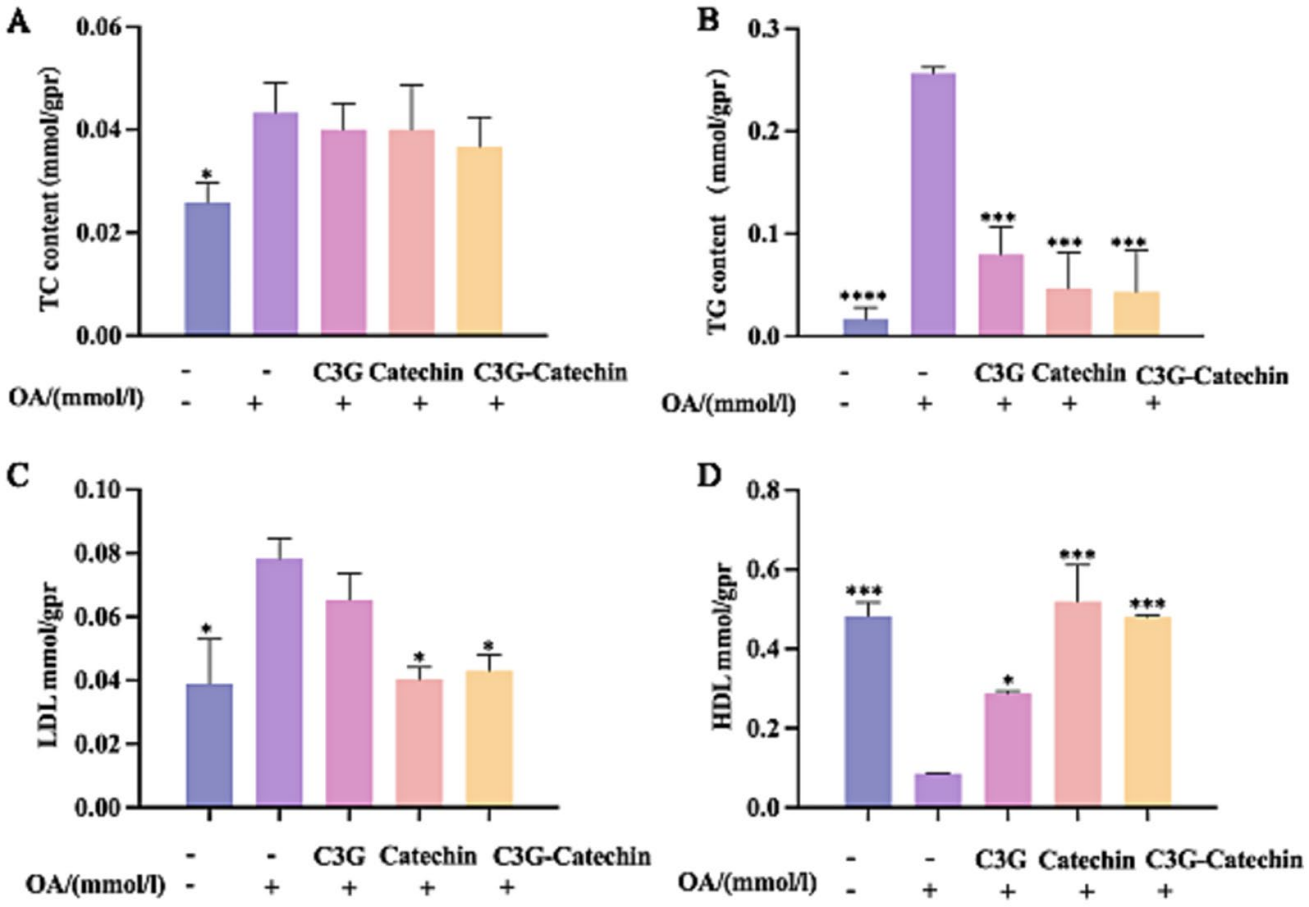
Characterization of the polyphenols’ lipid-lowering effects in the Caco-2/HepG2 co-culture model. ELISA was used to measure TC **(A)** and TG **(B)** in lysates from untreated, OA-induced, and compound-treated OA-induced co-culture HepG2 cells after the polyphenols had been absorbed and transported by Caco-2 cells. ELISAs for LDL **(C)** and HDL **(D)** were performed on lysates from the indicated treatments. Values are means + SD. *p < 0.05; ***p < 0.001; ****p < 0.0001. Data were obtained from Student’s *t* test.

The accumulation of fat in HepG2 cells was reduced by polyphenols, which are apparently transported passively through the small intestine and absorbed by Caco-2 cells. To further investigate their effects, we utilized a Caco-2/ HepG2 co-culture model that simulates the transport of polyphenols from the small intestine to the liver in mammals. TG and TC contents on the BL side were higher in HepG2 cells compared to the control; however, the accumulations of TG and TC were impeded in polyphenol-treated groups (Figures 4A,B). The reductions in TC content for the C3G, catechin and C3G-catechin treatment groups were 7.69, 7.69, and 15.38%, respectively, indicating an improvement in lipid deposition in liver cells following polyphenol treatment. Notably, TC content in the C3G-catechin group, which did not undergo intestinal transport, was reduced by 10%. Additionally, TG content decreased by 21% in the C3G group, indicating that the lipid-lowering effect of the polyphenols was more pronounced following intestinal transport, thus enhancing their bioavailability in HepG2 cells.

HDL-C is an important protective factor against metabolic syndrome, while LDL-C is often referred to as “bad” cholesterol (20, 21). After OA treatment, LDL-C levels increased significantly. Remarkably, this increase was reduced by 26.7% with C3G-catechin treatment (Figure 4C). However, C3G treatment did not change the LDL level. Although HDL-C levels decreased following OA treatment (Figure 4D), they increased with the addition of polyphenols. Notably, in the C3G-catechin group, HDL-C levels increased dramatically by 462% (5.6 fold) compared to those with OA treatment (Figure 4D). These findings indicate that polyphenols, particularly the C3G-catechin combination, have more significant hypolipidemic effects and exhibit a synergistic effect.

#### 3.4.2 Effects of polyphenols on the expression of lipid metabolism-related genes in the co-culture model

**Figure 5:**
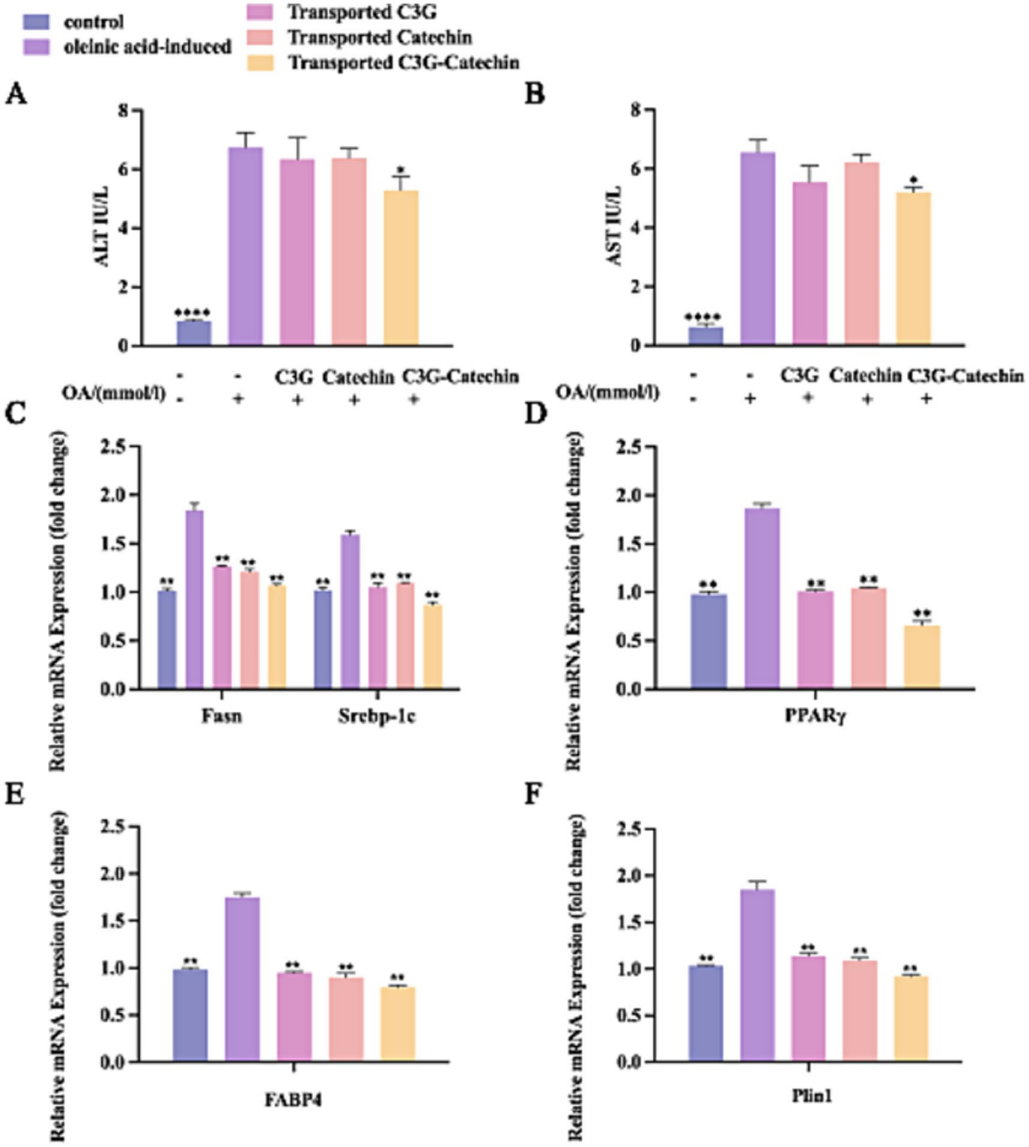
Polyphenols regulated lipid metabolism in the co-cultured cells. OA-induced Caco-2/HepG2 co-cultured cells were incubated with or without polyphenols, and lipid metabolism thereafter was measured by ELISA, RT-qPCR and Western blot. The effect of polyphenols on the contents of AST **(A)** and ALT **(B)** were evaluated. Transcriptional expression of Fasn, Srebp1-c **(C)**, PPARγ **(D)**, Fabp4 **(E)** and Plin1 **(F)** in lysates from untreated, OA-induced and polyphenols treated OA-induced co-culture HepG2 cells after polyphenols were absorbed and transported by Caco-2 cells. Values are means + SD. *p < 0.05; **p < 0.01; ****p < 0.0001. Data were obtained from Student’s *t* test.

Having observed that polyphenols enhanced HDL-C accumulation and reduced LDL-C accumulation in HepG2 liver cells, we further examined whether polyphenols influence the levels of AST and ALT, which are key indicators of liver health (22). An increase in ALT typically indicates acute hepatocellular damage (22). Our data showed that ALT levels significantly increased following OA induction (Figures 5A,B). After C3G-catechin combination treatment, the levels of AST and ALT were significantly reduced by 20.2 and 21.2%, respectively. However, this phenomenon was not observed from the individual treatment (C3G or catechin treatment). These data suggest that the combination exerts a more protective effect and demonstrates better synergistic interaction in the liver by significantly reducing AST and ALT levels in the cells.

Excessive fat accumulation can trigger a series of adverse metabolic effects. Nevertheless, specific factors produced by lipid cells regulate lipid accumulation, synthesis, transport, and storage (23). PPARγ, a ligand-activated transcription factor highly expressed in adipose tissue, plays an important role in regulating the gene expression involved in glucose and lipid metabolism (24). Srebp-1c, a sterol regulatory element-binding transcription factor, is essential for regulating intracellular lipid homeostasis and inducing the expression of biosynthetic enzymes for fatty acid synthesis, including fatty acid synthase (Fas) and acetyl-CoA carboxylase (Acc) (25). Recently, an inhibitor of fatty acid binding protein 4 (Fabp4) has been explored as a novel approach for treating obesity (25, 42). Previous studies have shown that perilipin 1 (Plin1) is highly expressed in mature white adipocytes and plays a role in regulating lipid breakdown. Moreover, Plin1 can activate the lipid droplet-associated protein Cidec/Fsp., facilitating lipid exchange, transfer, and droplet formation (26). Plin1 is highly expressed in obese individuals and is considered a significant factor in human obesity (27).

To assess whether polyphenols affect the transcriptional levels of genes related to lipid metabolism, we employed the q-PCR to analyze gene expression in the Caco-2/HepG2 co-culture model. This analysis aimed to explore the effects of polyphenols on regulating lipid synthesis, transport, and breakdown in the high-fat environment. Compared to the normal group, the expressions levels of lipid production-related transcription factors, including Srebp-1c, Pparγ, and Fasn, were significantly upregulated in the high-fat group (*p <* 0.01; Figures 5C,D). Notably, the expression levels of these factors were significantly downregulated (*p <* 0.01) following treatment with polyphenolic compounds. This effect was evident in the BL side cells after simulated intestinal absorption, indicating that the metabolites of absorbed polyphenols inhibited the intracellular expression of Pparγ, Fasn, and Srebp-1c. Furthermore, the inhibitory effect was more pronounced with C3G-catechin treatment. These data suggest that the functional activity and bioavailability of polyphenols are regulated upon absorption in the small intestine and that polyphenols may reduce lipid synthesis and uptake via the PPARγ-Fabp4/ PPARγ-Fasn pathway (28, 29), thereby decreasing lipid production and excessive accumulation, and exerting a lipid-lowering effect (30).

Further analysis revealed that the expression of Fabp4 was significantly upregulated following OA treatment compared to the control group (Figure 5E). However, with the addition of polyphenols, the expression levels were significantly downregulated (*p <* 0.01), suggesting that these polyphenols further suppressed FABPs expression in cells post-intestinal absorption. Similarly, OA enhanced the expression of Plin1 (Figure 5F), but polyphenols demonstrated inhibitory effects on Plin1 expression both before and after intestinal transport.

In summary, polyphenols including C3G, catechin, and the C3G-catechin combination, can inhibit the formation and accumulation of intracellular lipids by downregulating lipid synthesis-related transcription factors such as PPARγ, and genes associated with lipid production (Srebp-1c, Fasn), transport (Fabp4), and metabolism (Plin1). Among those, the C3G-catechin combination shows a better synergistic function on regulating fatty liver disease relevant factors.

#### 3.4.3 Translational regulation of hepatic lipid metabolism

**Figure 6:**
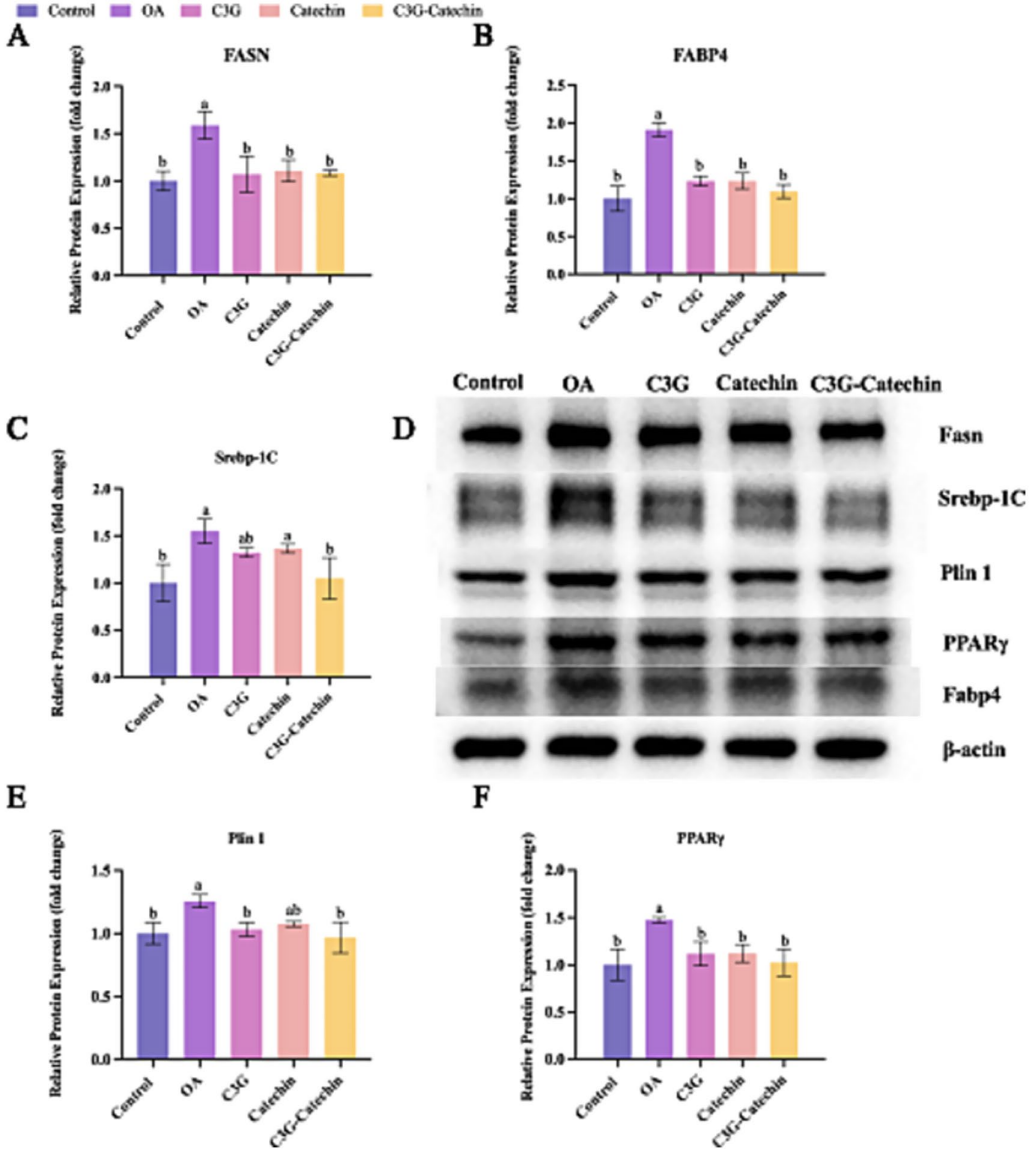
Translational regulation of liver lipid metabolism coordinated by polyphenols in the Caco-2/HepG2 co-culture system. **(A–C)** Densitometric quantification of Fasn **(A)**, Fabp4 **(B)** and Srebp-1c **(C)** protein levels in cells treated for 24 h with 50 μM of cyanidin-3-O-glucoside (C3G), catechin, C3G–catechin complex, respectively, or 1 mM oleic acid (OA). **(D)** Representative immunoblots for Fasn, Fabp4, Srebp-1c, PPARγ and Plin1 were obtained from the same experimental set. **(E,F)** Densitometric analysis of PPARγ and Plin1 protein expression, respectively. All densitometric values in panels **A–C** and **E,F** were derived from the immunoblots shown in panel D. Data were analyzed with SPSS 20.0 and are expressed as mean ± SD (*n* = 3 independent biological replicates). *p < 0.05, **p < 0.01 vs. the OA group.

Given our results, we further investigated whether polyphenols affect the translational regulation of hepatic lipid metabolism. We found that OA treatment significantly upregulated Fasn expression levels (OA: 1.59 ± 0.16-fold vs. control, *p* < 0.01), a hallmark of enhanced lipogenesis, validating our high-fat model (Figures 6A,D). Interestingly, C3G, catechin and their combination appeared to downregulate this process (C3G: 1.07 vs. OA, *p* < 0.05; Catechin: 1.11 vs. OA, *p* < 0.05; C3G-Catechin: 1.08 vs. OAl, *p* < 0.05, respectively) (Figures 6A,D), indicating that polyphenols can reduce the production of long-chain saturated fatty acids by downregulating fatty acid synthase. Additionally, Fabp4 expression levels were significantly reduced in the OA-induced high-fat group with the application of C3G, catechin or C3G-catechin (C3G: 1.23 vs. OA, *p <* 0.05; Catechin: 1.23 vs. OA, *p <* 0.01; C3G-Catechin: 1.09 vs. OA, *p* < 0.05, respectively; Figures 6B,D), suggesting that these polyphenols may regulate lipid trafficking and transport via fatty acid binding proteins. Given that lipid synthesis, trafficking and transport were regulated by polyphenols in high-fat cells, we speculated that lipid storage might also be affected. Indeed, the expression of Srebp-1c was reduced in the polyphenol-treated group compared to the control group (C3G: 1.32 vs. OA, *p <* 0.05; C3G-catechin: 1.05 vs. OA, *p* < 0.05, respectively; Figures 6C,D), indicating the involvement of polyphenols in lipid storage and demonstrating that the combination has a more significant effect on downregulating fat-related genes compared to the individual component.

Lipid storage primarily occurs within lipid droplets, which are implicated in hyperlipidemia and related metabolic diseases. We observed that Plin1 expression was reduced in the high-fat group with polyphenol treatments (C3G: 1.12 vs. OA, *p* < 0.05; Catechin: 1.14 vs. OA, *p <* 0.05; C3G-Catechin: 1.02 vs. OA, *p* < 0.05, respectively; Figures 6D,E), suggesting that polyphenols influence lipase access to stored lipids. Lastly, we investigated whether polyphenols regulate the upstream lipid metabolism signaling pathway by measuring PPARγ expression. Notably, PPARγ expression was significantly reduced (C3G: 1.03 vs. OA, *p* < 0.05; Catechin: 1.08 vs. OA, *p* < 0.05; C3G-Catechin: 0.97 vs. OA, *p* < 0.05, respectively; Figures 6D,F), showing that polyphenols negatively regulate lipid-related gene expression by downregulating PPARγ. These data align with previous transcriptional analyses and suggest that polyphenols may act as promising modulators of lipid homeostasis, offering therapeutic potential for metabolic disorders.

## 4 Discussion

Polyphenols, with their complex structural compositions, are widely present in fruits and vegetables and exhibit numerous biological activities, including antioxidant, anticancer, anti-inflammatory, cardioprotective, neuroprotective and antidiabetic properties. Polyphenols derived from rice anthocyanins and adzuki bean coat have been shown to improve lipid profiles in mammalian cells and exert a synergistic inhibitory effect on pancreatic lipase, a key enzyme involved in lipid hydrolysis related to obesity (16). Reducing fat absorption is crucial in anti-obesity strategies. This study showed that the intestinal absorption rate and membrane permeability of catechin were higher than those of C3G, consistent with previous studies suggesting a negative correlation between the degree of glycosylation and intestinal absorption (31, 43). Further investigation revealed that after intestinal absorption and transport, the effects of these polyphenols on lipid regulation in HepG2 cells were more pronounced. The enhanced effect, particularly for C3G, may be attributed to mechanisms such as enterohepatic circulation (EHC), which could potentially improve its bioactivity or cellular availability. However, it is important to note that our *in vitro* model (Caco-2/HepG2 co-culture/sequential treatment) does not fully encompass the complex *in vivo* physiology of EHC, which involves intricate interactions as substances travel between the intestine and liver via the systemic circulation, including the bloodstream. Therefore, this hypothesis remains speculative and requires validation using appropriate in vivo models (32).

Bad eating habits and inactive lifestyle have increased the prevalence of chronic metabolism disease (33), such as hyperlipidemia. Adherence to a healthy lifestyle that includes a high-quality diet, regular exercise, and adequate weight maintenance, is strongly associated with the fat-related disease management. Administration of a diet high in antioxidants which can be used to manage hyperlipidemia (34), can be sourced from legumes. Local food materials, such as legumes, that have high antioxidant content are black rice and red bean (16). Black rice and red bean are small, sweet-tasted, soft-textured and have anti-inflammatory response. However, few have been shown to have beneficial effects in animals when orally consumed, because of the poor bioavailability exhibited by most polyphenols following the ingestion (35). Consumed polyphenols by the body overcome various barriers during digestion and absorption, to reach the sites of action. Interestingly, polyphenols are also known to modify some of the metabolic and transport processes that govern bioavailability with side effects. Therefore, it is a chance to increase the bioactivity of beneficial polyphenols that improve their bioavailability by a simple synergistic reaction. Our study showed that C3G, catechin, and their combination-derived from black rice and red beans-significantly reduced TG and TC accumulation in HepG2 cells, aligning with previous research, which reported that extracts from various vegetables and fruits decrease lipid content in hepatocytes (36). Notably, the combination of C3G and catechin exhibited a synergistic effect, resulting in a more pronounced reduction in lipid accumulation compared to either compound alone. Our data further support the potential of these polyphenols in reducing lipid accumulation (37, 38).

Additionally, a study by Su et al. (39) demonstrated that a combination of hesperidin, nobiletin and tangeretin exhibited lipid-lowering activity, which was associated with decreased transcriptional levels of FAS. Suzuki et al. (40) reported that a highly absorbable catechin extract prevented diet-induced lipid-metabolism disorder in mice through marked inhibition of hepatic Srebp-1c expression, while Kim et al. (41) showed that (−)-epigallocatechin gallate blocked 3 T3-L1 adipocyte differentiation via the FoxO1/Srebp-1c axis. Consistent with these findings, our study also revealed that these polyphenols may exert their effects by inhibiting the transcriptional and translational expression of genes related to lipid synthesis, such as Srebp-1c, Pparγ, and Fasn.

This study comprehensively explored the anti-lipid accumulation effects and potential mechanisms of C3G, catechin, and their conjugate, C3G-catechin. Using human intestinal epithelial Caco-2 cells and HepG2 hepatoma cells as *in vitro* models, we examined the effects of these polyphenols on lipid accumulation. We elucidated the anti-obesity potential and possible mechanism in vitro, providing a theoretical basis for assessing their absorption, transport, and lipid-lowering properties. However, our study has some limitations. Notably, we primarily focused on an in vitro hepatic model without considering other potential sites of lipid accumulation, such as adipose tissue *in vivo*. Therefore, further research should investigate the effects of these polyphenols across different tissues and in various in vivo biological models.

## Data Availability

The datasets presented in this study can be found in online repositories. The names of the repository/repositories and accession number(s) can be found in the article/Supplementary material.

## References

[1] He Y Hu Y Jiang X Chen T Ma Y Wu S. Cyanidin-3-o-glucoside inhibits the UVB-induced ROS/COX-2 pathway in HaCaT cells. J Photochem Photobiol B. (2017) 177:24–31. doi: 10.1016/j.jphotobiol.2017.10.006, PMID: 29031211

[2] Cassidy A Rogers G Peterson JJ Dwyer JT Lin H Jacques PF. Higher dietary anthocyanin and flavonol intakes are associated with anti-inflammatory effects in a population of US adults. Am J Clin Nutr. (2015) 102:172–81. doi: 10.3945/ajcn.115.108555, PMID: 26016863 PMC4480670

[3] Min J Yu S-W Baek S-H Nair KM Bae O-N Bhatt A. Neuroprotective effect of cyanidin-3-O-glucoside anthocyanin in mice with focal cerebral ischemia. Neurosci Lett. (2011) 500:157–61. doi: 10.1016/j.neulet.2011.05.048, PMID: 21651957

[4] Wang S Li B Tan H Sun X Tian J Li S. Cyanidin-3-O-glucoside magic: unveiling the power to inhibit cholesterol absorption via the intestinal farnesoid X receptor–bile acids pathway with Lactobacillus marvel. Food Front. (2024) 5:2721–36. doi: 10.1002/fft2.482

[5] Wallace TC. Anthocyanins in cardiovascular disease. Advan Nutrition (Bethesda, Md). (2011) 2:1–7. doi: 10.3945/an.110.000042, PMID: 22211184 PMC3042791

[6] Bursill CA Roach PD. A green tea catechin extract upregulates the hepatic low-density lipoprotein receptor in rats. Lipids. (2007) 42:621–7. doi: 10.1007/s11745-007-3077-x, PMID: 17582541

[7] Prince PD Fischerman L Toblli JE Fraga CG Galleano M. LPS-induced renal inflammation is prevented by (−)-epicatechin in rats. Redox Biol. (2017) 11:342–9. doi: 10.1016/j.redox.2016.12.023, PMID: 28039839 PMC5200882

[8] Suzuki J-I Ogawa M Izawa A Sagesaka YM Isobe M. Dietary consumption of green tea catechins attenuate hyperlipidaemia-induced atherosclerosis and systemic organ damage in mice. Acta Cardiol. (2005) 60:271–6. doi: 10.2143/AC.60.3.2005003, PMID: 15999466

[9] Castell-Auví A Motilva MJ Macià A Torrell H Bladé C Pinent M. Organotypic co-culture system to study plant extract bioactivity on hepatocytes. Food Chem. (2010) 122:775–81. doi: 10.1016/j.foodchem.2010.03.052

[10] Yang M Lu X Xu J Liu X Zhang W Guan R. Cellular uptake, transport mechanism and anti-inflammatory effect of cyanidin-3-glucoside nanoliposomes in Caco-2/RAW 264.7 co-culture model. Front Nutr. (2022) 9:995391. doi: 10.3389/fnut.2022.995391, PMID: 36225868 PMC9549275

[11] Sadeghi Ekbatan S Iskandar MM Sleno L Sabally K Khairallah J Prakash S. Absorption and metabolism of phenolics from digests of polyphenol-rich potato extracts using the Caco-2/HepG2 co-culture system. Foods. (2018) 7:8. doi: 10.3390/foods7010008, PMID: 29329242 PMC5789271

[12] Teng Z Yuan C Zhang F Huan M Cao W Li K. Intestinal absorption and first-pass metabolism of polyphenol compounds in rat and their transport dynamics in Caco-2 cells. PLoS One. (2012) 7:e29647. doi: 10.1371/journal.pone.0029647, PMID: 22253753 PMC3258254

[13] Adom KK Liu RH. Rapid peroxyl radical scavenging capacity (PSC) assay for assessing both hydrophilic and lipophilic antioxidants. J Agric Food Chem. (2005) 53:6572–80. doi: 10.1021/jf048318o, PMID: 16104768

[14] Rafiei H Omidian K Bandy B. Dietary polyphenols protect against oleic acid-induced steatosis in an in vitro model of NAFLD by modulating lipid metabolism and improving mitochondrial function. Nutrients. (2019) 11:541. doi: 10.3390/nu11030541, PMID: 30832407 PMC6471211

[15] Liman M Wenji W Conghui L Haiyang Y Zhigang W Xubo W. Selection of reference genes for reverse transcription quantitative real-time PCR normalization in black rockfish (Sebastes schlegeli). Mar Genomics. (2013) 11:67–73. doi: 10.1016/j.margen.2013.08.002, PMID: 24007945

[16] Wang Y Chen L Liu H Xie J Yin W Xu Z. Characterization of the synergistic inhibitory effect of cyanidin-3-O-glucoside and catechin on pancreatic lipase. Food Chem. (2023) 404:134672. doi: 10.1016/j.foodchem.2022.134672, PMID: 36323025

[17] Chen J Lin H Hu M. Absorption and metabolism of genistein and its five isoflavone analogs in the human intestinal Caco-2 model. Cancer Chemother Pharmacol. (2005) 55:159–69. doi: 10.1007/s00280-004-0842-x, PMID: 15455178

[18] Chan KY Zhang L Zuo Z. Intestinal efflux transport kinetics of green tea catechins in Caco-2 monolayer model. J Pharm Pharmacol. (2007) 59:395–400. doi: 10.1211/jpp.59.3.0009, PMID: 17331343

[19] Zhang J Fan N Peng Y. Heat shock protein 70 promotes lipogenesis in HepG2 cells. Lipids in Health and Disease. (2018) 17. doi: 10.1186/s12944-018-0722-8 PMC5891916 29631603

[20] Ference BA Ginsberg HN Graham I Ray KK Packard CJ Bruckert E. Low-density lipoproteins cause atherosclerotic cardiovascular disease. 1. Evidence from genetic, epidemiologic, and clinical studies. A consensus statement from the European atherosclerosis society consensus panel. Eur Heart J. (2017) 38:2459–72. doi: 10.1093/eurheartj/ehx144, PMID: 28444290 PMC5837225

[21] Mani P Ren HY Neeland IJ McGuire DK Ayers CR Khera A. The association between HDL particle concentration and incident metabolic syndrome in the multi-ethnic Dallas heart study. Diabetes Metab Syndr Clin Res Rev. (2017) 11:S175–9. doi: 10.1016/j.dsx.2016.12.028, PMID: 27993539 PMC6190917

[22] Yao Y Li Z Qin B Ju X Wang L. Evaluation of the intracellular lipid-lowering effect of polyphenols extract from highland barley in HepG2 cells. Food Sci Human Wellness. (2024) 13:454–61. doi: 10.26599/fshw.2022.9250039

[23] Jump DB. Fatty acid regulation of hepatic lipid metabolism. Curr Opin Clin Nutr Metab Care. (2011) 14:115–20. doi: 10.1097/MCO.0b013e328342991c, PMID: 21178610 PMC3356999

[24] Ahmadian M Suh JM Hah N Liddle C Atkins AR Downes M. PPARγ signaling and metabolism: the good, the bad and the future. Nat Med. (2013) 19:557–66. doi: 10.1038/nm.3159, PMID: 23652116 PMC3870016

[25] Tung Y-C Hsieh P-H Pan M-H Ho C-T. Cellular models for the evaluation of the antiobesity effect of selected phytochemicals from food and herbs. J Food Drug Anal. (2017) 25:100–10. doi: 10.1016/j.jfda.2016.10.018, PMID: 28911527 PMC9333434

[26] Sun Z Gong J Wu H Xu W Wu L Xu D. Perilipin1 promotes unilocular lipid droplet formation through the activation of Fsp27 in adipocytes. Nat Commun. (2013) 4:1594. doi: 10.1038/ncomms2581, PMID: 23481402 PMC3615468

[27] Tai ES Ordovas JM. The role of perilipin in human obesity and insulin resistance. Curr Opin Lipidol. (2007) 18:152–6. doi: 10.1097/MOL.0b013e328086aeab, PMID: 17353663

[28] Che L Ren B Jia Y Dong Y Wang Y Shan J. Feprazone displays Antiadipogenesis and Antiobesity capacities in in vitro 3 T3-L1 cells and in vivo mice. ACS omega. (2021) 6:6674–80. doi: 10.1021/acsomega.0c05470, PMID: 33748580 PMC7970497

[29] Zhao Y Tang S Lin R Zheng T Li D Chen X. Deoxynivalenol exposure suppresses adipogenesis by inhibiting the expression of peroxisome proliferator-activated receptor gamma 2 (PPARγ2) in 3T3-L1 cells. Int J Mol Sci. (2020) 21:6300. doi: 10.3390/ijms21176300, PMID: 32878272 PMC7504378

[30] Bansode RR Randolph P Hurley S Ahmedna M. Evaluation of hypolipidemic effects of peanut skin-derived polyphenols in rats on Western-diet. Food Chem. (2012) 135:1659–66. doi: 10.1016/j.foodchem.2012.06.034, PMID: 22953907

[31] Gonzales GB Smagghe G Grootaert C Zotti M Raes K Van Camp J. Flavonoid interactions during digestion, absorption, distribution and metabolism: a sequential structure-activity/property relationship-based approach in the study of bioavailability and bioactivity. Drug Metab Rev. (2015) 47:175–90. doi: 10.3109/03602532.2014.1003649, PMID: 25633078

[32] Gui H Sun L Liu R Si X Li D Wang Y. Current knowledge of anthocyanin metabolism in the digestive tract: absorption, distribution, degradation, and interconversion. Crit Rev Food Sci Nutr. (2023) 63:5953–66. doi: 10.1080/10408398.2022.2026291, PMID: 35057688

[33] Haider N Abbas U Arif HE Uqaili AA Khowaja MA Hussain N. From plate to profile: investigating the influence of dietary habits and inactive lifestyle on lipid profile in medical students at clerkship. BMC Nutrition. (2024) 10:71. doi: 10.1186/s40795-024-00871-9, PMID: 38715144 PMC11077723

[34] Gong X Li X Xia Y Xu J Li Q Zhang C. Effects of phytochemicals from plant-based functional foods on hyperlipidemia and their underpinning mechanisms. Trends Food Sci Technol. (2020) 103:304–20. doi: 10.1016/j.tifs.2020.07.026

[35] Scheepens A Tan K Paxton JW. Improving the oral bioavailability of beneficial polyphenols through designed synergies. Genes Nutr. (2010) 5:75–87. doi: 10.1007/s12263-009-0148-z, PMID: 19841960 PMC2820202

[36] Mukherjee PK Nema NK Maity N Sarkar BK. Phytochemical and therapeutic potential of cucumber. Fitoterapia. (2013) 84:227–36. doi: 10.1016/j.fitote.2012.10.003, PMID: 23098877

[37] Ferré P Foufelle F. SREBP-1c transcription factor and lipid homeostasis: clinical perspective. Horm Res. (2007) 68:72–82. doi: 10.1159/000100426, PMID: 17344645

[38] Shimano H. SREBPs: physiology and pathophysiology of the SREBP family: physiology and pathophysiology of the SREBP family. FEBS J. (2009) 276:616–21. doi: 10.1111/j.1742-4658.2008.06806.x 19143830

[39] Su D Liu H Qi X Dong L Zhang R Zhang J. Citrus peel flavonoids improve lipid metabolism by inhibiting miR-33 and miR-122 expression in HepG2 cells. Biosci Biotechnol Biochem. (2019) 83:1747–55. doi: 10.1080/09168451.2019.1608807, PMID: 31017523

[40] Suzuki T Kumazoe M Kim Y Yamashita S Nakahara K Tsukamoto S. Green tea extract containing a highly absorbent catechin prevents diet-induced lipid metabolism disorder. Sci Rep. (2013) 3:2749. doi: 10.1038/srep02749 24067358 PMC3782887

[41] Kim H Hiraishi A Tsuchiya K Sakamoto K. (−) epigallocatechin gallate suppresses the differentiation of 3T3-L1 preadipocytes through transcription factors FoxO1 and SREBP1c. Cytotechnology. (2010) 62:245–55. doi: 10.1007/s10616-010-9285-x, PMID: 20596890 PMC2932904

[42] Cai H Liu Q Gao D Wang T Chen T Yan G. Novel fatty acid binding protein 4 (FABP4) inhibitors: virtual screening, synthesis and crystal structure determination. Eur J Med Chem. (2015) 90:241–50. doi: 10.1016/j.ejmech.2014.11.020, PMID: 25461324

[43] Dai JY Yang JL Li C. Transport and metabolism of flavonoids from Chinese herbal remedy Xiaochaihu-tang across human intestinal Caco-2 cell monolayers. Acta Pharmacologica Sinica. (2008) 29:1086–1093. 18718184 10.1111/j.1745-7254.2008.00850.x

